# Self-Healing Engineered Multilayer Coatings for Corrosion Protection of Magnesium Alloy AZ31B

**DOI:** 10.1021/acsmaterialsau.4c00170

**Published:** 2025-01-28

**Authors:** Mario Aparicio, Jadra Mosa, Miguel Gómez-Herrero, Zainab Abd Al-Jaleel, Jennifer Guzman, Mihaela Jitianu, Lisa C. Klein, Andrei Jitianu

**Affiliations:** † Instituto de Ceramica y Vidrio, Consejo Superior de Investigaciones Científicas (CSIC), Kelsen 5 (Campus de Cantoblanco), 28049 Madrid, Spain; ‡ Department of Chemistry, Lehman College, CUNY, Davis Hall, 250 Bedford Park Boulevard West Bronx, New York 10468, United States; § Department of Chemistry, William Paterson University, 300 Pompton Road, Wayne, New Jersey 07470, United States; ∥ Department of Materials Science and Engineering, Rutgers University, 607 Taylor Road, Piscataway, New Jersey 08854, United States; ⊥ Ph.D. Program in Chemistry and Biochemistry, The Graduate Center of the City University of New York, 365 Fifth Avenue, New York, New York 10016, United States

**Keywords:** Self-healing coating, Hybrid glass, Cerium (III) ion doping, Sol−Gel process, Corrosion protection, Magnesium alloys

## Abstract

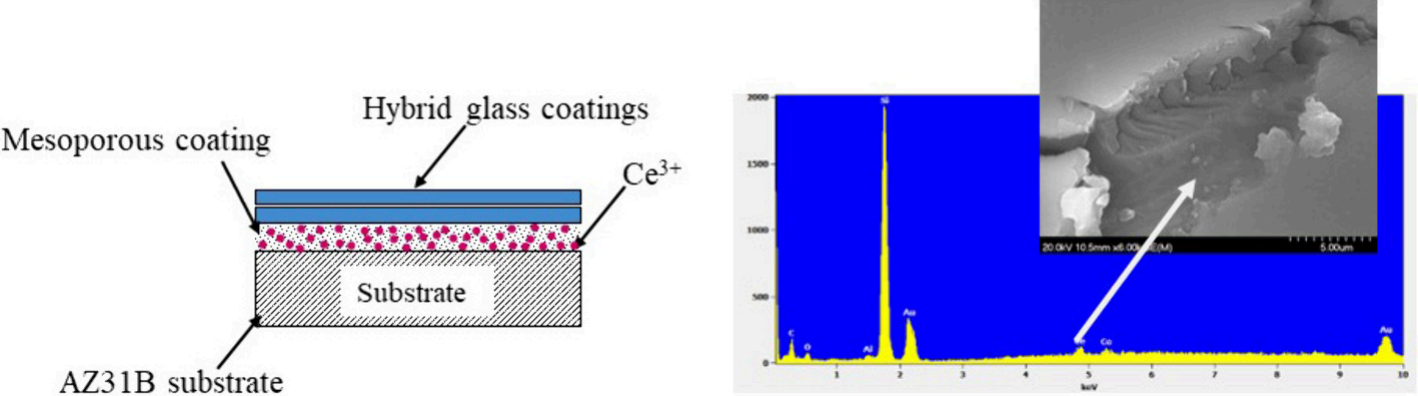

Nonporous, crack-free hybrid glass coatings have provided excellent corrosion protection to the AZ31B magnesium alloy. However, if a crack develops in the coatings, then corrosion will proliferate at that point. The novelty of this study consists of engineering a bilayer protection system that combines the “barrier” properties of the hybrid glass coatings with the “inhibitor” or “self-healing” effect of an internal layer of mesoporous silica doped with cerium(III) ions. The mesoporous layer was obtained using a sol–gel solution with 1 mol % cerium(III) ions. The inner cerium-doped mesoporous coating has a thickness of 0.25 μm, and the electrochemical characterization through Open circuit potential (OCP) and Electrochemical Impedance Spectroscopy (EIS) indicates a corrosion inhibition process provided by cerium(III) ions triggered by the corrosion. The combination of the Ce-doped and hybrid glass coatings reaches a total thickness of 5.1 μm. The corrosion evaluation through OCP and EIS does not show any evidence of corrosion during the first 575 h of immersion. After this, there are several steps of a sudden drop in potential and subsequent recovery of the previous values, which could be associated with the activation of the corrosion inhibition mechanism provided by the Ce (III) ions. EIS show a maximum impedance module of 10^6.7^ Ohm cm^2^, a decrease of impedance values and phase angle fluctuations after the potential drops observed, and, then, a recovery of the previous values of impedance and phase angle. This behavior confirms activation of the corrosion inhibition mechanism. Polarization curves shows that the multilayer coating leads to a low current density (∼10^–11^ A cm^–2^), around 5 orders of magnitude lower in comparison with the bare substrate. A *post-mortem* SEM-EDX analysis study, performed on the cracks generated during electrochemical testing, shows the accumulation of cerium as a consequence of the corrosion inhibitory process.

## 1 Introduction

Due to the necessity to reduce fuel consumption and CO_2_ emissions, magnesium alloys, with a very low density (1738 kg/m^3^) compared to steel and aluminum alloys, are attractive for aerospace and the automotive industry. Another desired application is for medical prosthetics since magnesium has almost the same density as human bones.^1,2^ The main disadvantage of magnesium is its high surface reactivity, especially with water. From the thermodynamic point of view, due to the low standard potential, magnesium is an active metal prone to corrosion in a humid environment.^3−5^

The stability of magnesium can be increased by passivation alloying with different metals such as aluminum – AZ alloys,^6^ zinc,^7^ copper – CMg1,^8^ manganese – AM505,^9^ nickel –Mg-0.6Ni,^10^ chromium Mg–Cr,^11^ and titanium Mg_80_–Ti_20_.^12^ Beside magnesium, aluminum is the second most abundant element used in the AZ magnesium-type alloys. The presence of aluminum determines the formation of oxides, hydroxides, and carbonates on the surface of magnesium alloys which prevent further degradation increasing the corrosion resistance.^13−16^

Another approach to improve stability of magnesium toward corrosion is using corrosion inhibitors such as sodium benzotriazole, Ce (III) ions,^17,18^ organic coatings,^19^ phosphate coatings,^20^ cerium phosphate-based additive in hybrid epoxy-silane coatings,^21^ perfluorinated polysiloxane coatings with graphene oxide,^22^ sol–gel coatings, etc.

Inorganic and hybrid organic–inorganic sol–gel protective coatings have been proposed for protection against corrosion of AZ-group Mg alloys, but thermal treatments at relatively high temperatures for coating densification were, in some cases, mismatched with the requirement for preservation of the microstructure and properties of the magnesium alloy protected substrate. On the other hand, the presence of residual defects, such as cracks and porosity, resulted from high volumetric shrinkage of the sol–gel coating, limiting their corrosion protection during long exposure times to aggressive electrolytes.^23−31^ Correa et al. used methyltriethoxysilane coatings doped with Ce(III) ions to prevent the corrosion of AZ91 alloy.^32^ They showed that the corrosion resistance is proportional to the concentration of Ce(NO_3_)_3_ which provides active protection. However, a high concentration of Ce (III) ions in the methyltriethoxysilane film led to the degradation of the coatings due to modification of the siloxane network. Zanotto et al. used a modified ORMOSIL with 3-mercapto-propyl-trimethoxysilane alongside Ce(III) ions to protect AZ31.^33^ Their successful active barrier was attributed to lower porosity, to fewer defects, and to the self-healing ability provided by Ce(III) ions.

Some strategies to improve the “barrier” properties of the sol–gel coatings are based on crack sealing,^27^ the use of cross-linkers,^28,31^ curing agents,^29^ amino acids,^30^ combination with inhibitor-doped coatings,^18,19,34,35^ or incorporating corrosion inhibitors in the electrolyte.^36^

Historically poly(benzylsilsesquioxane) was the first system for which the melting gels were first reported,^37^ followed by studies on melting gels prepared using phenyltriethoxysilane (PhTES) and diphenyldiethoxysilane (DPhDES) or methyltriethoxysilane (MTES) and dimethyldiethoxysilane (DMDES).^38−43^ A specific characteristic of these hybrid organic–inorganic melting gels which was not identified for other sol–gel materials, is that they are rigid at room temperature, become fluid at a temperature T_1_ (∼110 °C), and can be resoftened over and over again as long the temperature of consolidation T_2_ was not reached. Nevertheless, after consolidation at a temperature T_2_ (T_2_ > T_1_) (135–170 °C), the gels are consolidated and transformed into hybrid glasses. Despite their name, “melting gels” do not present a classical melting process. These materials are organically modified polysilsesquioxanes with low glass transition temperatures^44^ and softening points.^39−43^ These properties can be associated with the organic groups involved, which plays a critical role in the formation of the organically modified polysilsesquioxanes structures.^42,43,45^ The main factors include steric hindrance and increasing hydrophobicity between the organic moieties. The formation of irreversible hybrid glasses take place at consolidation temperature T_2_ due to the cross-linking of the silica chains into three-dimensional networks.^44,46^ Increasing the temperature increases the mobility of the organically modified polysilsesquioxanes chains, which leads to a decrease of the steric hindrance and an increase in the rate of polycondensation reactions of the silicon-bonded alkoxy and/or hydroxyl groups. Before reaching the temperature of consolidation T_2_ (135–170 °C), the process of softening (110 °C) - becoming rigid (room temperature) – resoftening (110 °C) can be recurring many times as needed. This property was used with great results in the processing technique of obtaining protective hybrid glass coatings.^40,44−47^

The versatility of the processability of the melting gels allows these to be used primarily as anticorrosive coatings.^48−51^ Thick (>500 μm) hybrid glass coatings on AISI 304 Stainless Steel were obtained by the consolidation of the methyltriethoxysilane (MTES) and dimethyldiethoxysilane (DMDES) melting gels.^48^ For these coatings, polarization and impedance measurements in NaCl solution indicate a very durable surface during four months of immersion. Current densities are 6 orders of magnitude lower in comparison to the bare substrate. On the other hand, one of the biggest challenges is to obtain anticorrosive thin coatings. For the same systems, coatings were prepared with thicknesses up to 10 μm using diluted melting gels.^49^ The 70 mol % MTES – 30 mol % DMDES coating gives the best behavior for corrosion protection of the AISI 304 in NaCl solutions. Potentiodynamic polarization results of this coating show a good barrier film with a passive range of 500 mV and a very low anodic current density of 3 × 10^–11^ A cm^–2^ after two months of immersion in the electrolyte, a reduction of more than 4 orders of magnitude in comparison with the stainless-steel reference.

Due to excellent results obtained on AISI 304, we refocused our attention on more sensitive substrates such as magnesium-based alloy AZ31B which requires corrosion protection. Using the same MTES-DMDES melting gels, uniform, and crack-free hybrid glass coatings were obtained on the AZ31B surface.^50^ For this study, two different thickness ranges around 1000 and 10–56 μm were investigated. The 56 μm coatings were obtained by depositing two thin layers on top of each other. For this case, there was spectroscopic evidence for a chemical interaction between the hybrid glass coatings and the substrates, showing the presence of Si–O–Mg bonds, which can explain the excellent adhesion of the coatings to the substrates. The hybrid glass coating consisting of two thin layers, 56 μm, provides the best corrosion resistance. Corrosion characterization presents very low current densities (10^–13^ A cm^–2^), impedance of 10^10^ Ohm cm^2^, and one time constant associated with the coating.

Furthermore, thick hybrid glass coatings of ∼1000 μm were obtained with phenyl trimethoxysilane (PhTMS) alongside diphenyl dimethoxysilane (DPhDMS) and used to protect titanium alloy substrates to decrease their corrosion in acid media.^51^ For this system, it was demonstrated that the hardness of the hybrid glass coatings varies with the content of methanol used during melting gels synthesis. These papers aimed to develop defect-free coatings, avoiding the typical shrinkage of the classical sol–gel coating during the densification treatment. These nonporous and crack-free hybrid glass coatings showed very good corrosion protection in NaCl solution because they behave as an excellent “barrier”. However, if a crack develops in the coating due to physical impact or a defect produced during the processing, corrosion will develop rapidly at that point, triggering dangerous localized pitting. As these hybrid glass coatings obtained from the melting gels are nonporous,^52^ self-healing agents are entrapped in the coatings preventing their mobility and consequently their ability to heal the corrosion sites.

**Figure 1:**
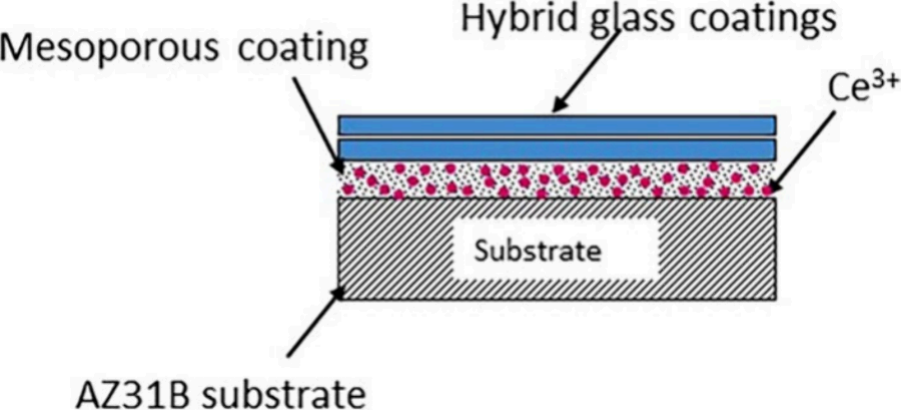
Schematic of the engineered self-healing coatings.

This work focuses on trying to overcome this situation through engineering a bilayer protection system that combines the “barrier” effect of melting gel/hybrid glass coatings with the “inhibitor” effect embedded in an internal layer of mesoporous silica doped with cerium(III) ions. Cerium(III) ions due to their ability to oxidize to Ce (IV), have previously demonstrated the ability to reduce the corrosion process of metals through their rapid diffusion and reaction in cathodic zones with the consequent precipitation of very stable cerium oxides and hydroxides.^32,34−36,53−56^ However, the protection offered by cerium(III) ions is usually limited due to the presence of too many defects (pores and cracks), where the cerium(III) has to act as a healing agent. This work intends to improve the inhibition efficiency of a cerium-based coating by combining it with a coating of melting gels/hybrid glasses, inherently free of defects. More specifically, here we engineered a multilayer structure where the magnesium AZ31B substrate is in direct contact with a mesoporous silica coating, which has a dual role. First it acts as a reservoir where the Ce(III) ions are deposited, and second, it allows the mobility of Ce(III) ions to access the corrosion sites. The Ce (III) ions entrapped in the mesopores are followed by the deposition of a bilayer of hybrid glass protective barrier coatings obtained by consolidating the melting gels as illustrated in Figure 1. The role of the mesoporous coating is to provide a space for storage and membrane for mobility of the cerium(III) ions for the self-healing functionality.

Therefore, this work aims to achieve a combination of barrier and inhibitor properties provided by hybrid glass and cerium(III)-doped coatings, respectively. Further, increasing the stability of the protective system with immersion time would be relatively simple by increasing the thickness of hybrid glass coating as shown in our previous papers, focused solely on the barrier functionality.^50^ We envision that this engineered complex barrier can be used for the automotive and aerospace industry.

## 2 Experimental Section

### 2.1 Synthesis Procedure and Preparation of Coatings

The coatings used in this study were prepared in two steps. In the first step, a cerium(III)-doped mesoporous coating was prepared and deposited on the magnesium substrate, followed by the deposition of a melting gel followed by their consolidation and transformation into a hybrid glass coating.

#### 2.1.1 Synthesis Procedure and Preparation of the Ce-Doped Silica Mesoporous Coating

The mesoporous coatings were prepared using tetramethyl orthosilicate (TMOS), 98% (Millipore-Sigma, Burlington, MA), cerium(III) nitrate hexahydrate (Ce(NO_3_)_3_·6H_2_O), 99.5% (Millipore-Sigma, Burlington, MA), anhydrous ethanol (Millipore-Sigma, Burlington, MA), and cetyltrimethylammonium bromide (CTAB), (Millipore-Sigma, Burlington, MA). All the chemicals were used as received without conducting any further purification. In the standard procedure, the molar ratios between reactants were TMOS (1) - CTAB (0.16) - EtOH (35) - H_2_O (5) - Ce(NO_3_)_3_·6H_2_O (1). The sol was prepared by adding 1.9 g of CTAB to 54 mL of ethanol with continuous stirring for 5 min at room temperature. To this solution, 5.2 g of TMOS was added and continued to be stirred for 5 min. Further, 3 mL of water was added to the system. The pH of the deionized water was lowered to pH 1.5 using 1 M HCl (Fisher Scientific, Atlanta, GA). The hydrochloric acid was used as a catalyst to promote the hydrolysis/polycondensation reactions. The so prepared solution was stirred in a closed system for 1 h at room temperature. Then, 3.3 g of cerium (III) nitrate hexahydrate was added into the solution to have a concentration 1 mol % of Ce (III) ions. The solution was continuously stirred for an additional 1 h, and the final solution was allowed to age for 24 h at room temperature.

In this study, the substrates were AZ31B magnesium alloy with a size of 3.0 cm × 2.5 cm x 0.10 cm, acquired from Magnesium Elektron North America Inc. (Madison, IL). Before deposition of the coatings, the substrates were manually polished using 30 μm 400 grit, 10 μm 800 grit, 5 μm 1200 grit, 5 μm, and 1 μm 3 M (St. Paul MN) premium SiC abrasive discs until a mirror-like surface was obtained on both faces. The substrates were washed with absolute ethanol and dried. The mesoporous coatings containing cerium were deposited using an MTI Desktop Dip Coater (Richmond, CA, USA) with an adjustable speed. The support was immersed by vertically dipping in the 24 h’ age solution and maintained for 1 min. Then, this was withdrawn with a speed of 16.2 cm min^–1^ required to create uniform coatings. The coating was dried at room temperature for 1 h and then for 48 h at 60 °C to remove the excess ethanol from pores, followed by the annealing at 110 °C for 1 h, where excess water present was removed. The cooled coatings were washed with warm ethanol to remove the template CTAB surfactant. After drying at room temperature, the coatings were thermally treated at 60 °C for 2 h to remove the excess of ethanol followed by heating at 150 °C for 24 h for condensation of the siloxane matrix. Soon after the temperature was increased to 200 °C for 5 min to consolidate the pores without their collapse followed by the final thermal treatment at 250 °C for 13.5 h, where all the organic components were removed.

The Ce(III) silica doped sol remained after the deposition was allowed to gel at room temperature and was dried at 60 °C for 48 h. The dry powder was crushed using an agate mortar. Then it was thermally treated in the same way as the coating followed by washing with ethanol to remove the CTAB. After that the powder was heated to 150 °C for 24 h followed by heating at 200 °C for 5 min and a final thermal treatment at 250 °C for 13.5 h.

#### 2.1.2 Synthesis Procedure and Preparation of the Melting Gel Coatings

Melting gel preparation with methyltriethoxysilane and dimethyldiethoxysilane was reported previously.^46^ However, a brief description of the melting gel preparation used in this study is presented here. The precursors for melting gel synthesis were methyltriethoxysilane (MTES) (Millipore-Sigma, Burlington, MA), a monosubstituted alkoxide, and dimethyldiethoxysilane (DMDES) (Millipore-Sigma, Burlington, MA), a disubstituted alkoxide. For this synthesis, we used as catalysts hydrochloric acid (37.4%) (Fisher Scientific, Atlanta, GA) and ammonia (∼30%) (Millipore-Sigma, Burlington, MA). As solvent was employed anhydrous ethanol (Millipore-Sigma, Burlington, MA). All reagents were used without further purification. The molar composition of the melting gel used in this study is 70 mol % MTES – 30 mol % DMDES. The synthesis of the 70%MTES-30%DMDMS melting gels had three stages. First, the initial solution was prepared by mixing water with hydrochloric acid and then half of the alcohol. Using the other half of the ethanol and MTES a second solution was prepared. Under constant stirring (∼500 rpm), the second solution was added dropwise into the first one. This solution, for prehydrolysis, was continuously stirred at room temperature for 3 h in a sealed beaker. The molar ratios of the reagents used MTES: EtOH: H_2_O: HCl were 1:4:3:0.01. In the second stage of the synthesis, the disubstituted alkoxide (DMDES) was diluted with ethanol using a molar ratio of DMDES: EtOH = 1:4. This solution was added dropwise into the MTES: EtOH:H_2_O: HCl initial mixture. This reaction mixture was kept under constant magnetic stirring (∼500 rpm) in a sealed beaker at room temperature for two additional hours, while the reagents underwent hydrolysis and polycondensation reactions. Lastly, in the third stage, the second catalyst, ammonium hydroxide, was added dropwise to the reaction mixture. The molar ratio of (MTES + DMDES):NH_4_OH was 1:0.01. After this, the solution was stirred for one more hour in the sealed beaker and then for 48 h at room temperature in an open beaker until gelation occurred. The so prepared gel was thermally treated at 70 °C for 17 h to allow the evaporation of residual ethanol. During gelation, NH_4_Cl was formed as a byproduct. This is not soluble in organically modified polysilsesquioxanes. Dry acetone is a good solvent to decrease the viscosity of the polysilsesquioxanes, also being chemically inert toward these and easy to remove via evaporation. Furthermore, the NH_4_Cl is not soluble in the acetone. Taking advantage of the fact that acetone will lower the viscosity of the organically modified polysilsesquioxanes and will not dissolve the NH_4_Cl, dry acetone was used to facilitate the filtration of the viscous polysilsesquioxanes. For this 10 mL of dry acetone (Spectranal, Millipore-Sigma, Burlington, MA) were added into the organically modified polysilsesquioxanes and homogenized by stirring at 500 rpm After 30 min of homogenization the solution was filtered under vacuum. The supernatant was kept and transferred to another beaker and stirred with a speed of ∼600 rpm for ∼6 h until all acetone was evaporated and it gelled once again. The final MTES-DMDES gel was thermally treated at 70 °C for 24 h. The purpose of this thermal treatment was to remove all traces of acetone and ethanol. This was followed by another thermal treatment at 110 °C for the removal of unreacted water. At this point, a transparent colorless melting gel was obtained. For the preparation of “barrier” thin coatings, the melting gel was diluted with absolute ethanol, using an ethanol/gel ratio of 10.160*g*/7.746g. A homogeneous solution of the melting gels in ethanol was obtained in ∼36 h under constant shaking.

The substrates coated with the cerium-doped mesoporous silica coatings were then coated with the diluted melting gel by using the same dip coating procedure. The support was immersed by vertically dipping in the diluted melting gel solution and maintained for 1 min followed by withdrawn with a speed of 10.0 cm min^–1^. The coating was kept for 1 h at room temperature and then thermally treated at 140 °C for 17 h. This temperature was determined previously to be the temperature of formation of the hybrid glasses from the melting gels with 70 mol % MTES – 30 mol % DMDES composition.^46^ After cooling, the procedure was repeated in order to obtain a bilayer protection system.

### 2.2 Structural and Electrochemical Characterization

Thermogravimetric and Differential Thermal Analysis (TG-DTA) was done under air flow of 100 mL min^–1^ and at a heating rate of 5 °C min^–1^ between 50 and 1000 °C (TG- DTA; S2 EXSTAR 6000, TG/DTA6200, Seiko, Japan). The surface area of the powder matrix samples was analyzed by the Brunauer-Emmet-Teller (BET) method using a BET surface analyzer (Micromeritics, Tristar II 3020). The samples were degassed in the presence of nitrogen at 100 °C for 24 h, and then the measurement was carried out by absorption/desorption of a nitrogen/helium mixture. FTIR spectra were recorded between 4000 cm^–1^ to 400 cm^–1^ for 100 cycles per scan with a resolution of 4 cm^–1^ using an IS-10 Nicolet spectrometer (Thermo Scientific Waltham, Massachusetts) equipped with a Smart Endurance ATR attachment (diamond crystal). Evaluation of the coating integrity and chemical composition was done using a field-emission scanning electron microscope (HITACHI S-4700) equipped with energy-dispersive X-ray spectroscopy (NORAN System SIX). The coating thickness was measured on the cross-section of the coated samples. The coated metal substrate was cut, placed in a mold, and covered with epoxy resin. After unmolding, the cross-section of the samples was polished, and a thin layer of gold was deposited by sputtering before electron microscope observations. A post-mortem evaluation (morphology and chemical composition) of the sample surface after electrochemical tests was performed using the same equipment. Coating adhesion to the magnesium substrate was evaluated using microscratch tests (model APEX-1, CETR equipment). A “progressive load” mode, i.e., increasing the load while the tip moves, was performed from zero to 125 mN. This test allows us to know the load in which the coating begins to crack and delaminate, estimating the adhesion of the coating. The microscratch tests were accomplished using a conical-type diamond with a 5-μm tip radius and a 3 mm scratch. Normal load applied (Fz) and tangential force (Fx) vs horizontal displacement (Y) were recorded. The residual scratch pattern was studied by the same SEM microscope to relate Fz and Fx values to the morphology of the scratch made on the coating under the application of the progressive load.

Electrochemical tests were conducted at room temperature in 0.35 wt % NaCl solutions using a potentiostat (SP-200, Bio-Logic SAS) and a three-electrode glass cell. The coated metal was positioned vertically to minimize the effect of the corrosion products. A 7.0 cm^2^ Pt mesh was used as counter electrode, a saturated calomel electrode (SCE) as reference electrode, and coated samples (0.785 cm^2^) as working electrode. All tests were performed at least twice for each sample. Potentiodynamic polarization curves at 0.16 mV s^–1^ of the magnesium alloy protected with the multilayer coating and the bare alloy after one h of immersion in 0.35 wt % NaCl solution were performed to evaluate the “barrier” functionality of the coatings. Open Circuit Potential (OCP) and Electrochemical Impedance Spectroscopy (EIS) were also performed in the same tested area. EIS measurements were accomplished with sweeping frequencies from 20,000 to 10^–2^ Hz, modulating 0.050 V (rms) around the open circuit potential. In this case, the objective is to evaluate not only the barrier effect provided by the coating but also the corrosion inhibition effects delivered by cerium(III) ions.

## 3 Results and Discussion

### 3.1 Ce (III)-doped Silica Mesoporous Coating

**Figure 2:**
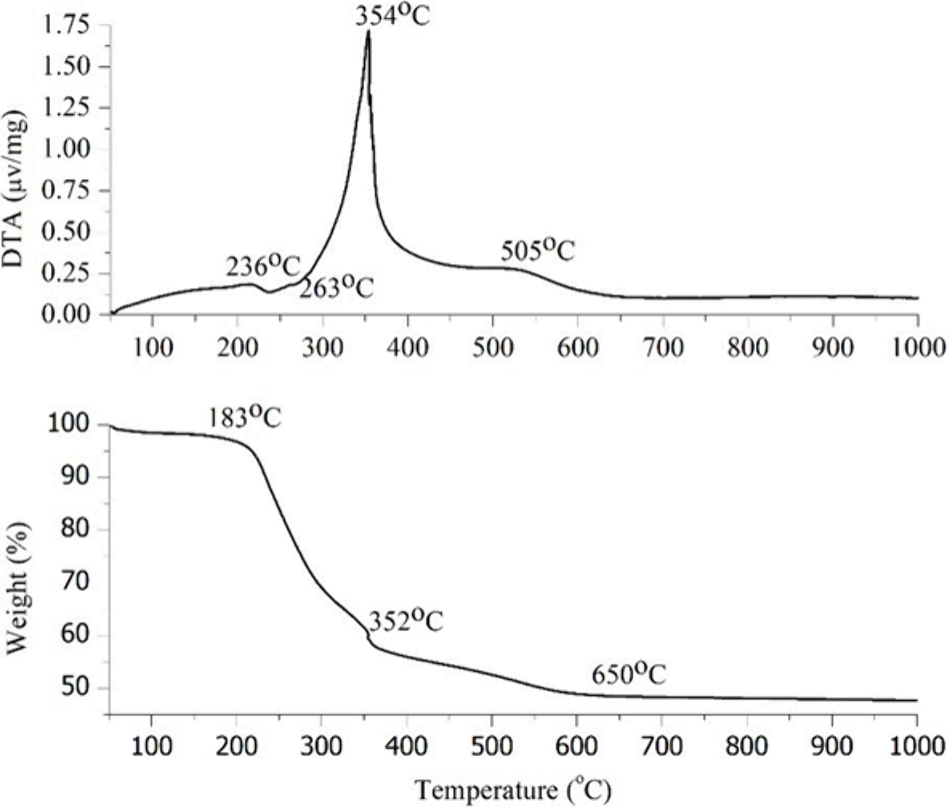
TG-DTA analysis of cerium-doped mesoporous silica powder.

The thermal stability was investigated using thermogravimetric analysis coupled with differential thermal analysis (TG- DTA). These are illustrated in Figure 2.

The thermal decomposition of the cerium-doped mesoporous silica powder, as prepared, occurred in two steps. The first step of decomposition is happening between 180 and 300 °C with an exothermal effect on the DTA curve at ∼236 °C. This step corresponds to the decomposition of the organic constituents such as the rest of methoxy groups. Additionally, at 246 °C was detected a small endothermic effect which can be assigned to the decomposition of the CTAB. The second step of decomposition was observed between 320 and 650 °C. This step had two exothermal effects at 354 and 505 °C. The first exothermal effect can be assigned to the decomposition of NO_3_^–^ ions while the effect from 505 °C can be assigned to the decomposition of the rest of the CTAB.

**Figure 3:**
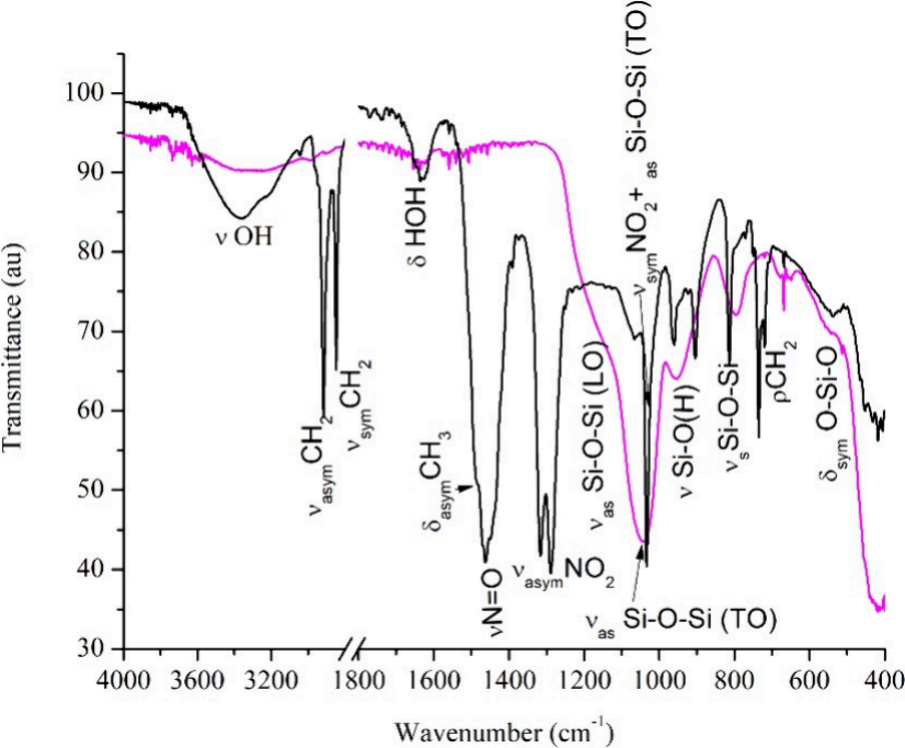
FTIR before and after the washing/thermal treatment at 250°C.

The powder obtained in the same way as the coatings was washed with ethanol to remove the CTAB and thermally treated identically as the coatings at 150 °C for 24 h followed by heating at 200 °C for 5 min and a final thermal treatment at 250 °C for 13.5 h, where most of the organic components were removed as it was observed in the FTIR spectra (Figure 3).

Figure 3 displays the FTIR spectra of the powders before and after the washing/thermal treatment. The samples before washing show the presence of the CTAB and nitrate ions, while the sample after the washing and thermal treatment shows that all the surfactant CTAB was successfully removed, as well as the nitrate ions. The final spectrum shows the presence of all of the characteristic bands for ≡Si–O–Si≡ and ≡Si–OH vibrations and the presence of the hydroxyl groups bonded to the silica network. The specific vibration for the presence of Ce–O bond should be at ∼450 cm^–1^. Here, there is overlap with δ Si–O–Si which appear in the same region of the spectrum.

**Figure 4:**
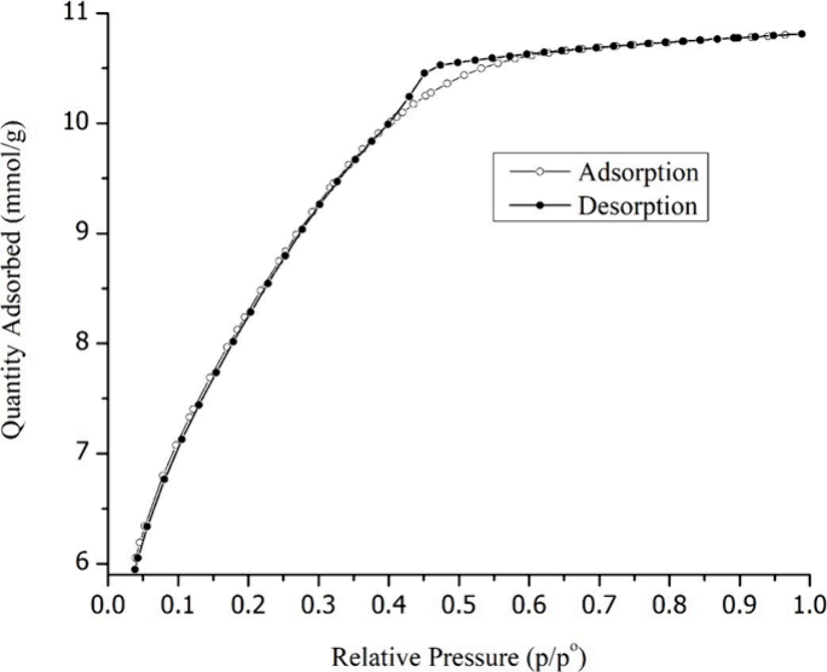
Adsorption–desorption isotherms of cerium-doped mesoporous silica.

The adsorption–desorption isotherms of the samples obtained under the same conditions as those for the coatings are presented in Figure 4.

This is a type IV isotherm that is typical for the mesoporous materials. The BET surface area was measured to be 635.7 m^2^ g^–1^, and the BJH adsorption average pore diameter (4 V/A) calculated is 2.497 nm.

**Figure 5:**
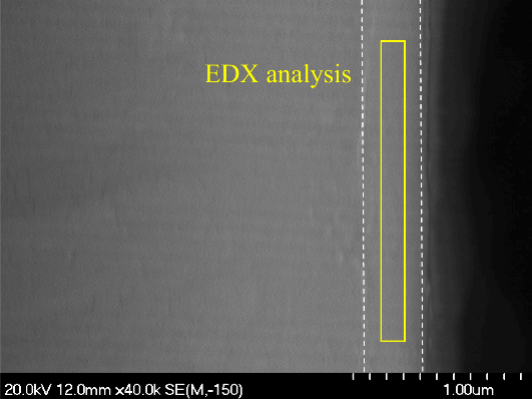
Cross-section SEM images of magnesium substrate coated with cerium-doped silica mesoporous coating; inset shows the area for EDX analysis.

Figure 5 presents a scanning electron microscopy micrograph of the cross-section of a magnesium substrate coated with the cerium-doped silica mesoporous coating.

The image shows a homogeneous layer with a thickness of around 0.25 μm. EDX analysis (Figure S1 and Table S1) confirms the presence of silicon, cerium, and oxygen in the layer and other elements from the magnesium alloy.

The SEM analysis of the fresh surface of cerium-doped silica mesoporous coating (Figure S2) shows at low magnifications (X500) that the coating has adapted well to the surface of the metal substrate, reproducing the machining lines of the magnesium alloy. Precipitates typical of the alloy and some cracks due to the roughness of the metal substrate and the low thickness of the internal coating are observed. At higher magnifications (X6000) it is observed in greater detail how the coating adheres well to the roughness of the magnesium alloy. Increasing the magnification further (X50000), the microcracking of the coating can be distinguished. This morphology is not suitable to offer a good barrier against the electrolyte, but it is an adequate structure for the movement of cerium ions to provide a self-healing function. The incorporation of the hybrid coating homogeneously covers the porous morphology of the cerium-doped silica mesoporous coating (Figure S3) without evidence of defects.

**Figure 6:**
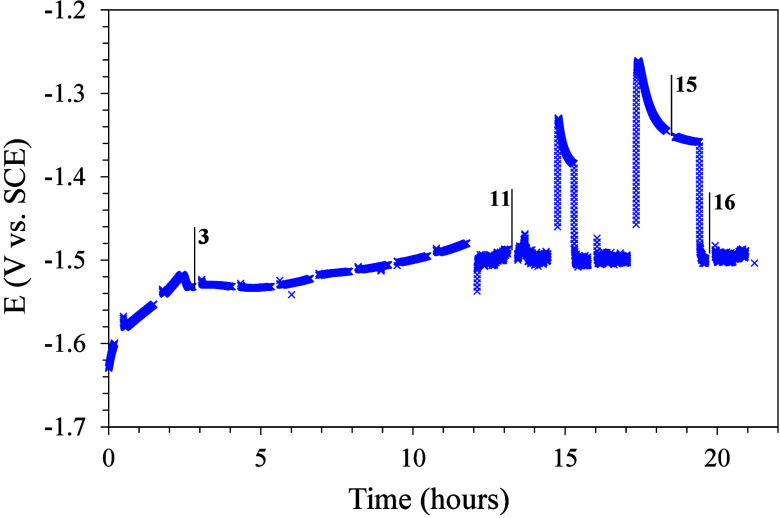
Open circuit potential (OCP) vs immersion time of magnesium substrate coated with the cerium-doped silica mesoporous coating.

Figure 6 presents the corrosion potential variation with the immersion time of the metal substrate coated with a cerium-doped silica mesoporous coating.

**Figure 7:**
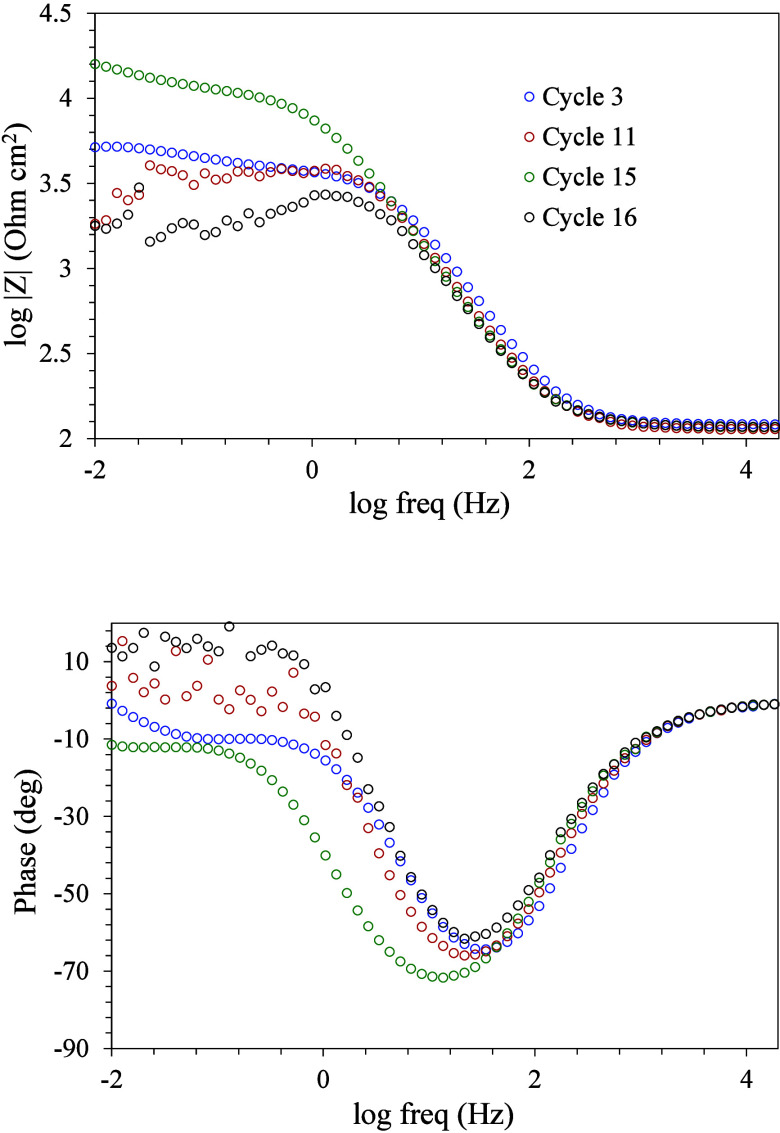
Electrochemical Impedance Spectroscopy (EIS) measurements at different immersion times of a magnesium substrate coated with cerium-doped silica mesoporous coating.

The curve presents a gap at different times associated with the impedance measurements that were performed. Specific EIS tests indicated with their cycle numbers in this plot, are shown in Figure 7. The results of Figure 6 show an initial period of stability (12 h of immersion) without significant fluctuations in potential values. Considering the low thickness of this coating and the presence of mesoporosity, it can be assumed that there is some degree of corrosion but that it is not extensive during this initial period. After this period, potential fluctuations increase at around −1.5 V vs SCE, likely associated with a more intense corrosion process. In addition, there are two potential jumps at 15 and 18 h of immersion toward less negative values that later return to −1.5 V vs SCE. This behavior may be associated with a corrosion inhibition process provided by cerium(III) ions triggered by the extensive corrosion. As expected, this mechanism is limited for this single thin coating considering its physical characteristics and the high reactivity of the magnesium substrate.

The four impedance measurements marked on the potential–time plots are shown in Figure 7.

The EIS 3 measurement presents the typical results of the first stages of the corrosion process of a magnesium alloy with one time constant at medium frequencies related to the charge transfer of the corrosion process, and another time constant at low frequencies associated with ion diffusion through the corrosion product layer.^33^ However, in the case of curve 11, already in the period of the greatest fluctuation of the potential values, a reduction of the impedance values and a high dispersion of the phase values at low frequencies are observed, which are associated with more intense corrosion of the substrate. Subsequently, curve 15 presents a significant change, considerably increasing the total impedance and stabilizing again the phase values. This behavior could be associated with the inhibition effect of cerium ions after the self-healing mechanism is activated by the increase in the corrosion level of the substrate. After a short period of self-protection, the potential falls again, and curve 16 shows an extensive corrosion behavior.

An EDX analysis study was carried out in different areas of the sample surface after the electrochemical tests. The results were compared with those obtained with the untested coating. The mean value of the Si/Ce atomic ratio for the bare coating surface is 20.8 (Figure S4). On the other hand, the average Si/Ce value obtained by analyzing the corrosion products is around 1.4 (Figure S5, Point 1), while the value in the surrounding area around the corrosion products and at different distances is in the range between 4.5 and 14.3 (Figure S5, Points 2 and 3). These results seem to indicate a diffusion of cerium ions from untested coating areas toward the area where the corrosion products are located. The high concentration of cerium ions in the corrosion products could be related to a mechanism of active corrosion inhibition. The initiation of the corrosion process activated this mechanism, causing the diffusion of cerium ions and the reaction of the hydroxyl groups produced in the cathodic reaction from the reduction of oxygen and water, generating cerium hydroxides/oxides. The precipitation of these cerium compounds in the corrosion zones reduces the corrosion rate of the metal substrate, accounting for the sudden changes in the open-circuit potential, impedance, and phase angle values.

The corrosion of the magnesium alloy AZ31B in a neutral medium generates a local increase in alkalinity.^57^ The increase in the concentration of OH^–^ groups at the active corrosion points produces the diffusion of cerium ions and their subsequent reaction. This process is rapid as can be observed in Figure 6 of the variation of the open circuit potential versus the immersion time. The appearance of the corrosive process generates a sudden increase in the potential and an immediate progressive decrease due to the inhibitory effect of the cerium ions.

### 3.2 Combination of Ce (III)-Doped and Hybrid Glass Coatings

**Figure 8:**
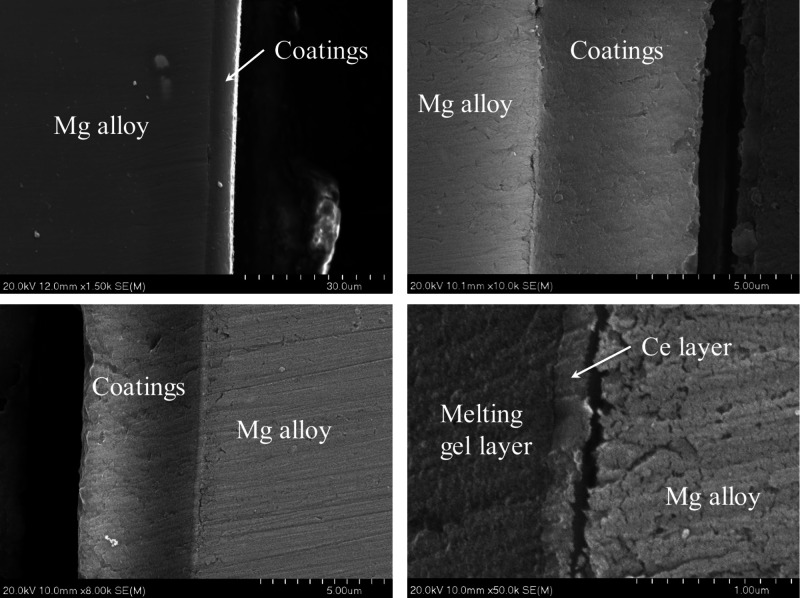
Cross-section SEM images of a magnesium substrate protected with the multilayer coating.

Figure 8 shows different scanning electron microscopy images of the cross-section of the metallic substrate protected with a multilayer coating.

**Figure 9:**
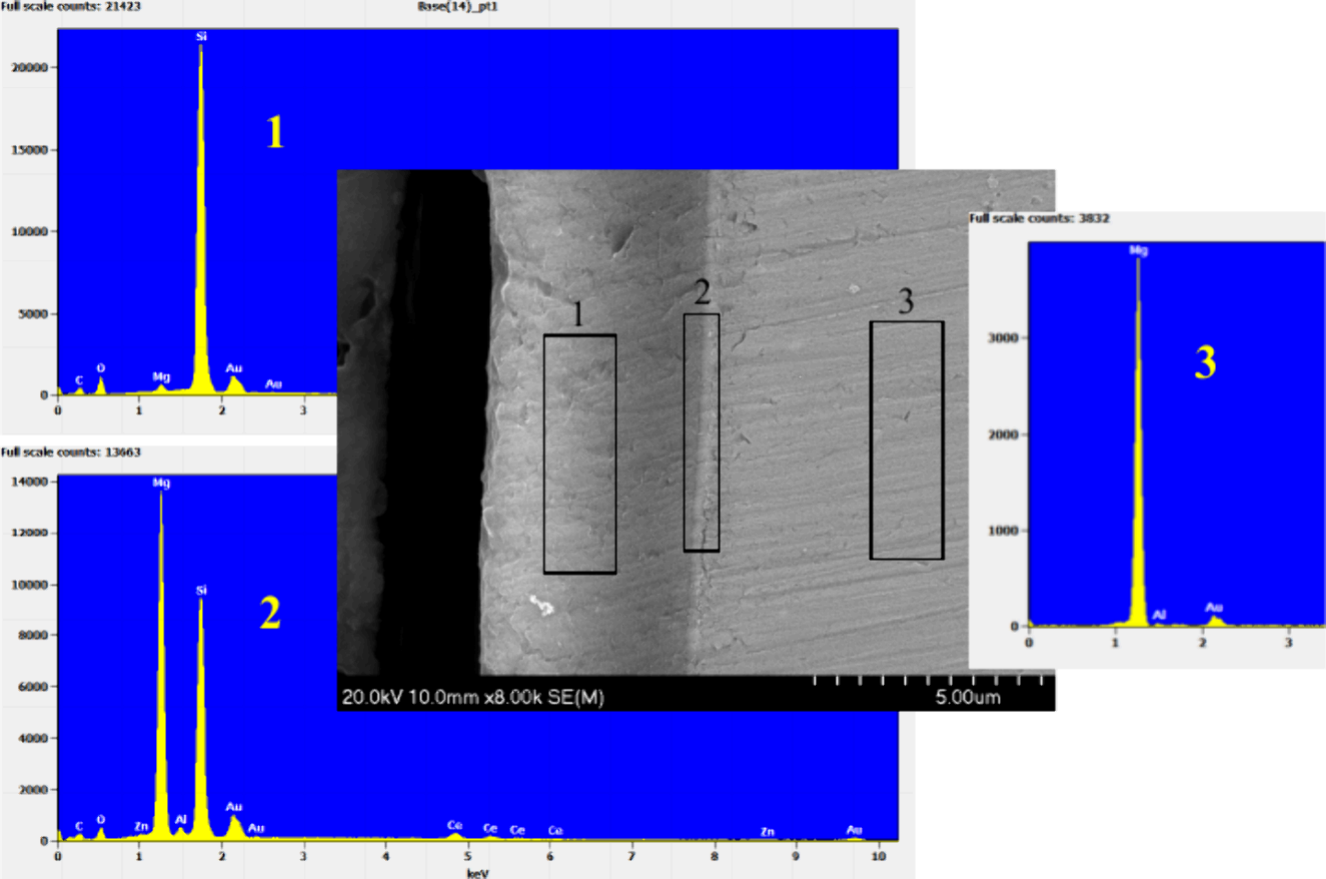
EDX analysis of a magnesium substrate protected with the multilayer.

As the coatings are prepared by dip coating, both sides of the substrate have similar layers. The thickness of the multilayer coating is around 5.1 μm. This thickness corresponds mainly to the bilayer coating of hybrid glass since, as can be seen in the image at higher magnifications, the internal coating of Ce-doped silica has a thickness of around 0.2 μm. The identification of both types of layers and the magnesium substrate is confirmed by EDX analysis (Figure 9 and Table S1), showing the presence of magnesium, silicon, and cerium as the main elements.

The FT-IR spectrum of the bilayer hybrid glass coating (Figure S6) shows only the presence of the final hybrid coating. The spectrum shows the presence of the characteristic vibrations of the methyl groups bonded on the ≡Si–O–Si≡ backbone, in addition to the typical Si–C vibrations due to the presence of H_3_C–Si≡ bonds.

**Figure 10:**
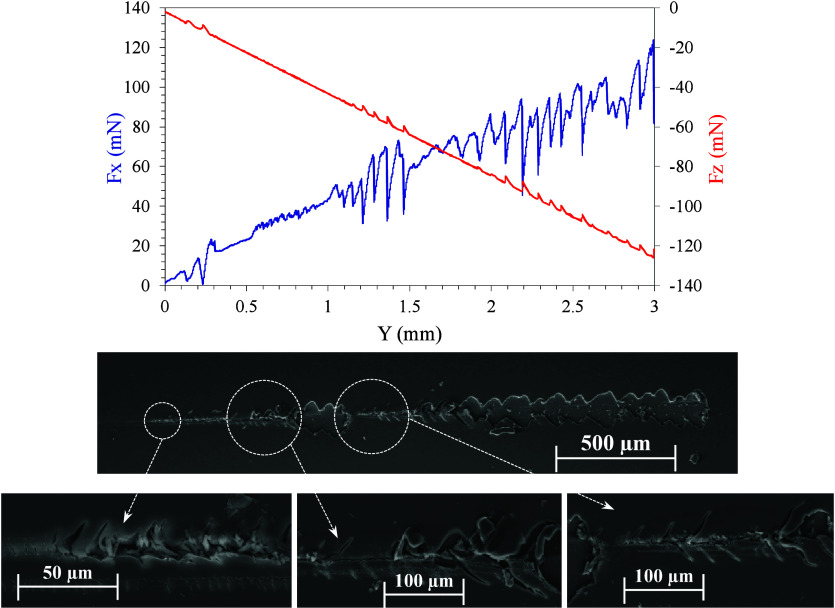
Microscratch test of the bilayer coating: normal load (Fz) and tangential force (Fx) vs horizontal displacement (Y), and SEM images of the scratch pattern.

Figure 10 presents the microscratch results with the normal load applied (Fz), tangential force (Fx), and surface SEM images of the scratch pattern on the bilayer coating. The load application begins on the left side of the overall SEM image and increases as it moves toward the right.

Initially, below 30 mN, the indenter produces only transverse cracks. At higher loads, it is observed in the SEM images how a deep groove is generated in the coating without delamination. When the value of around 45 mN is reached, the first delamination zone occurs due to the entrained material. Once this piece of layer is separated, no delamination is observed until reaching 70 mN, from which the delamination is generalized. These results indicate adequate adhesion of the coating that would allow subsequent processing, such as the application of paints.

**Figure 11:**
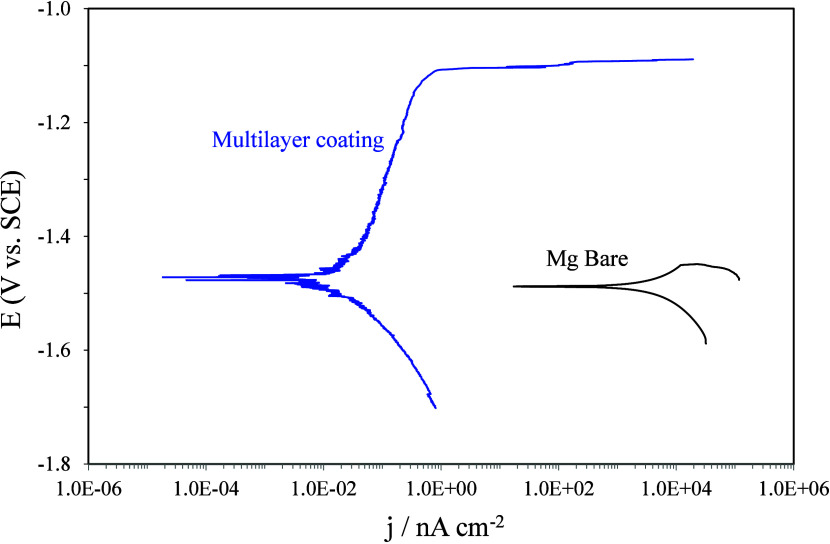
Polarization tests of the coated substrate and the bare substrate after 1 h of immersion in the NaCl solution.

Figure 11 shows the potentiodynamic polarization curve of the magnesium alloy protected with the multilayer coating and the bare alloy after one h of immersion in 0.35 wt % NaCl solution.

The bare magnesium alloy has mild protection as a result of the presence of oxides on the surface, while the coated substrate has stronger corrosion protection. The multilayer coating leads to a low current density (∼10^–11^ A cm^–2^), around 5 orders of magnitude lower in comparison to the bare substrate. The breakdown potential increases up to −1.1 V vs SCE, clear evidence of a very stable coating.

**Figure 12:**
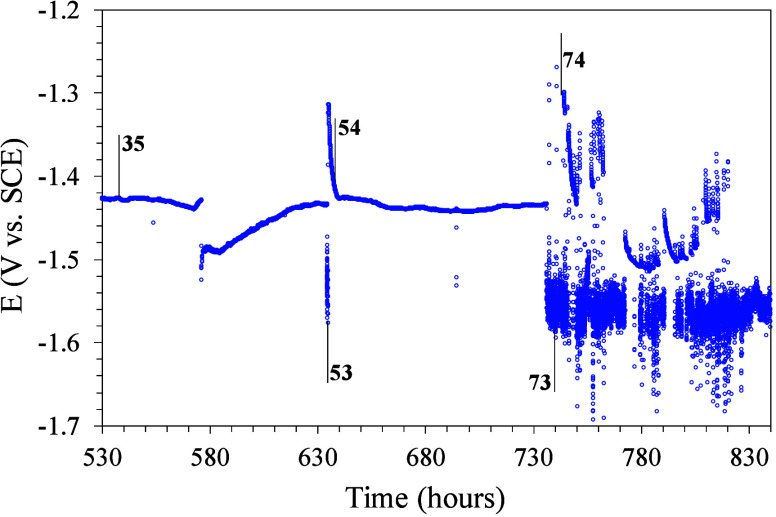
Open circuit potential (OCP) versus immersion time of magnesium substrate coated with a multilayer coating.

**Figure 13:**
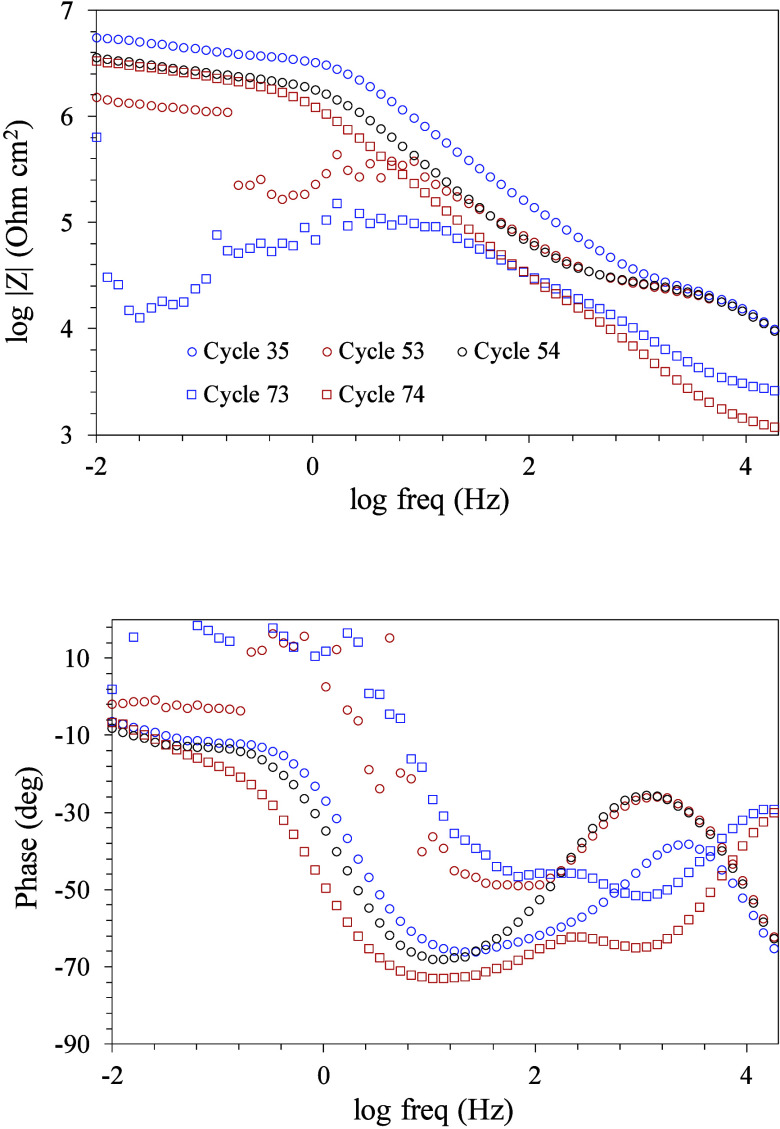
Impedance measurements (EIS) at different immersion times of the metal substrate protected with the multilayer coating.

Figure 12 presents the corrosion potential changes with immersion time for the multilayer coating. In addition, also in this case, the curve shows gaps at different times associated with the impedance measurements performed. Specific EIS measurements (Bode plots), indicated with cycle numbers in this plot, are shown in Figure 13.

Figure 12 only shows the results after 530 h of immersion since before there were no significant changes in the potential value because of the powerful barrier effect provided by the outer bilayer of hybrid glass. Until reaching 575 h of immersion, there are no significant changes in potential and the values do not fluctuate considerably, indicating that the corrosion up to this point is not extensive. The EIS 35 measurement, indicated in the graph, is representative of this area. At 575 h, there is a sudden drop in potential and a subsequent and progressive recovery of the previous values, which could be associated with a more intense corrosion process and the initiation of the active corrosion inhibition mechanism provided by the cerium (III) ions from the bottom layer. After 635 h of immersion, there is a new drop and recovery of the potential value, faster in this case. The EIS 53 and 54 tests have been carried out after the drop in potential and after the recovery of the previous value, respectively. Next, the figure shows a prolonged period of stability in the potential values until reaching 735 h of immersion, at which there is a new drop-in potential and a new recovery (EIS 73 and 74 tests). From this moment, there are successive stages with reduction and increase in potential, but already with large fluctuations, which seems to indicate that the combination of the “barrier” and “inhibitor” effects offered by the coatings is already limited at these immersion times.

As already indicated, the EIS 35 measurement (540 h of immersion) in Figure 13 is representative of the stability zone of the open circuit potential before the fluctuations caused by the corrosion process.

The curves show four time constants that can be associated with the hybrid glass coating (≥10^4.3^ Hz), Ce (III)-doped mesoporous silica coating (10^2.7^ Hz), charge transfer of corrosion process (10^1.3^ Hz), and ion diffusion (≤10^–1^ Hz). The maximum value of the impedance module reached at 10^–2^ Hz is 10^6.7^ Ohm cm^2^, which indicates considerable corrosion resistance. The EIS 53 measurement (635 immersion hours), after the sudden drop in the open circuit potential, shows significant changes. The time constant associated with the melting gel coating (≥10^4.3^ Hz) remains unchanged, indicating that the corrosion process has not damaged this outer layer at this moment. At lower frequencies, the curves diverge from the previous one, showing an irregular time constant at 10^1.7^ Hz assigned to the charge transfer of the corrosion process and the Ce (III)-doped silica coating. The value of the phase angle of this time constant is lower than the previous one, an unequivocal sign of a more intense corrosive process. At lower frequencies, fluctuation of the impedance and phase values is observed, reaching a maximum impedance value of 10^6.2^ Ohm cm^2^, which is associated with a reduction of the impedance associated with the charge transfer. EIS 54 measurement, only one h after the EIS 53 measurement, shows the almost total recovery of the previous values of impedance and phase angle, showing again the four-time constants that were observed in the EIS 35 measurement. The maximum value of the impedance of the EIS 54 measurement (10^6.5^ Ohm cm^2^) increases relative to the previous one, and it is close to the value of the EIS 35 measurement. The electrochemical behavior observed in this measurement with an increase in the open circuit potential and recovery of the impedance and phase angle values are associated with the initiation of the active corrosion inhibition mechanism described above.

EIS 73 and 74 measurements are additional examples of the intensification of the corrosion process and the immediate initiation of the active corrosion inhibition mechanism, respectively. The EIS 73 measurement shows some differences concerning measurement 53, also taken after worsening of the corrosive process. In this case, the high-frequency time constant associated with the melting gels/hybrid glass coating has shifted to 10^3.1^ Hz, reducing the associated value of the impedance modulus and phase angle, indicating the gradual deterioration of the outer coating. At frequencies lower than 10^2^ Hz, the values are similar to those of the EIS 53 measurement with data fluctuation because of the intense corrosive process. The EIS 74 measurement shows again the clear recovery of the impedance and phase angle values after activation of the inhibition mechanism. It is observed that the high-frequency time constant associated with the outer coating is similar to the previous one (EIS 73 measurement), indicating that there is no improvement in this coating, since it is far from the surface of the metallic substrate and is not affected by the activation of the inhibition mechanism. However, despite the gradual deterioration of the coatings, the high efficiency of the inhibition process provided by the cerium ions makes it possible to recover the maximum impedance values, bringing them closer to the initial ones.

According to some related studies where the barrier and self-healing functionalities have been compared, for example, Zhang et al.^19^ developed for corrosion protection of AZ31 magnesium alloy a microarc oxidation (MAO)/epoxy resin (EP) composite coatings. Mesoporous silica nanocontainers with sodium benzoate (SB) inhibitors were incorporated into the MAO and EP layers. The thickness of the coatings (120 μm) provided a good barrier functionality, reaching impedance modulus of 10^10^ Ohm cm^2^ after 90 days of immersion in NaCl solutions. The embedded nanocontainers in the MAO coating seem to play a sealing role in the micropores, preventing further permeation of the aggressive medium. However, no evidence of self-healing behavior is detected in the EIS results. Instead, a gradual degradation and reduction of impedance are observed with the immersion time. In contrast, our results show clear evidence of a self-healing functionality after the penetration of the electrolyte reaching the metal substrate.

Another approach to reduce the corrosion of AZ31 magnesium was using coatings produced by growth of the corrosion inhibitors intercalated in layered double hydroxide (Mg–Al LDH) and then sealing it by a hydrophobic coating.^18^ Authors claim that this composite achieves excellent corrosion inhibition. Although impedance values of 10^7^ Ohm cm^2^ at initial immersion time are obtained, no impedance measurements have been performed with immersion time to evaluate the self-healing functionality. An alternative study combining a cerium conversion layer with a sol–gel hybrid coating based on tetraethoxysilane (TEOS) and (3-glycidoxypropyl) trimethoxysilane (GPTMS) for corrosion protection of WE43 magnesium alloy shows self-healing effect using a local electrochemical impedance spectroscopy experiment in the mapping mode (LEIM).^34^ These experiments indicate that Ce species from the underlying conversion layer can migrate to defect sites and hinder the development of corrosion activity. However, the maximum impedance values achieved are limited (below 10^5^ Ohm cm^2^) without showing evidence of a self-healing effect. These results are probably associated with coating defects (pores and fissures) as shown by electron microscopy images, common in traditional sol–gel coatings. This is the main difference in comparison with our coatings, which despite using similar precursors, did not provide for the removal of solvents and water during the thermal treatment of the coatings avoiding the presence of defects.

Figure S7 shows an SEM image of an example of the cracks generated during electrochemical testing, as well as two magnifications to be able to appreciate the details. EDX analyses have been performed on the crack, and the results indicate the accumulation of cerium despite the high thickness of the outer layers as a consequence of the corrosion inhibitory process.

## 4 Conclusions

A bilayer smart protection system combining the “barrier” effect of hybrid glass coatings with the “inhibitor” effect of an internal layer of mesoporous silica doped with cerium(III) ions has been developed. This engineered self-healing barrier can be used in the automotive or aerospace industry. This process is potentially cost-effective, since the cost of replacement of the entire magnesium-based part might be more expensive than a pretreatment. In fact, the pretreatment is designed to increase the lifetime of the part or device even in cases of penetration of the protective barrier. The morphological and structural analysis of the cerium(III)-doped mesoporous coating displays a 0.25 μm average thickness with all of the characteristic bands for Si–O vibrations and hydroxyl groups bonded to the silica network (FTIR). Adsorption–desorption isotherms evaluation show a type IV isotherm, characteristic of mesoporous materials, with a surface area of 635.7 m^2^ g^–1^, and a BJH adsorption average pore diameter (4 V/A) of 2.497 nm. The electrochemical characterization of this Ce (III)-doped coating indicates a corrosion inhibition process that was provided by cerium ions. The initiation of the corrosion process automatically activated this mechanism. Cerium(III) ions reacted with hydroxyl groups produced by the reduction of oxygen and water during the cathodic reaction. The reaction produced cerium hydroxides/oxides. A *post-mortem* SEM-EDX analysis confirms the migration of cerium(III) ions from untested coating areas toward the region of the corrosion products. As expected, this mechanism is limited for this single thin coating, considering its porous structure and the high reactivity of the magnesium substrate. The combination of Ce (III)-doped and hybrid glass coatings produces a total thickness of 5.1 μm. The layer shows good adherence to the magnesium substrate as measured in microscratch tests.

Polarization curves show that the multilayer coating leads to a low current density (∼10^–11^ A cm^–2^), around 5 orders of magnitude lower in comparison with the bare substrate.

The corrosion evaluation through the OCP and EIS does not show any evidence of corrosion during the first 575 h of immersion in the electrolyte because of the powerful barrier effect provided by the outer hybrid glass coating. After this immersion time, there are several steps of a sudden drop in potential and subsequent recovery of the previous values, which could be associated with a more intense corrosion process and activation of the active corrosion inhibition mechanism provided by the cerium ions from the bottom layer. EIS results show a maximum impedance module of 10^6.7^ Ohm cm^2^. The measurements at different immersion times indicate a decrease of impedance values and phase angle fluctuations after the potential drops observed and, then, a recovery of the previous values of impedance and phase angle. This behavior confirms the activation of the corrosion inhibition mechanism described above. A *post-mortem* SEM-EDX analysis study, performed on the cracks generated during electrochemical testing, shows the accumulation of cerium as a consequence of the corrosion inhibitory process.

## Supporting Information Available

EDX analysis of the cerium-doped silica mesoporous coating before electrochemical testing; SEM images of the fresh surface of cerium-doped silica mesoporous coating and for the multilayer coating; postmortem SEM and EDX analysis of the cerium-doped silica mesoporous coating and multilayer coatings; FTIR analysis of the multilayer surface; element concentration from EDX analysis (PDF)
