# Supplementary material for: Self-Healing Engineered Multilayer Coatings for Corrosion Protection of Magnesium Alloy AZ31B
Source: ACS Mater Au. 2025 Jan 28;5(2):409–20. doi: 10.1021/acsmaterialsau.4c00170 (PMC11907293; doi:10.1021/acsmaterialsau.4c00170)
Supplement: Supplementary file 1 — mg4c00170_si_001.pdf [file mg4c00170_si_001.pdf]

Supplementary Information

Self-healing Engineered Multilayers Coatings for Corrosion protection of Magnesium alloy AZ31B

Mario Aparicio [a,*], Jadra Mosa [a], Miguel Gómez-Herrero [a], Zainab Abd Al-Jaleel [b], Jennifer Guzman [b], Mihaela Jitianu [c] , Lisa C. Klein [d], Andrei Jitianu [b,e,*]

[a] Instituto de Ceramica y Vidrio, Consejo Superior de Investigaciones Científicas (CSIC), Kelsen 5 (Campus de Cantoblanco), 28049 Madrid, Spain

[b] Department of Chemistry, Lehman College, CUNY, Davis Hall, 250 Bedford Park Boulevard West Bronx, New York 10468, United States

[c] Department of Chemistry, William Paterson University, 300 Pompton Road, Wayne, New Jersey 07470

[d] Department of Materials Science and Engineering, Rutgers University, 607 Taylor Road, Piscataway, New Jersey 08854, United States

[e] Ph.D. Program in Chemistry and Biochemistry, The Graduate Center of the City University of New York, 365 Fifth Avenue, New York, New York 10016, United States

***Corresponding authors.**

E-mail addresses: maparicio@icv.csic.es *(Mario Aparicio),* ANDREI.JITIANU@lehman.cuny.edu *(Andrei Jitianu)*

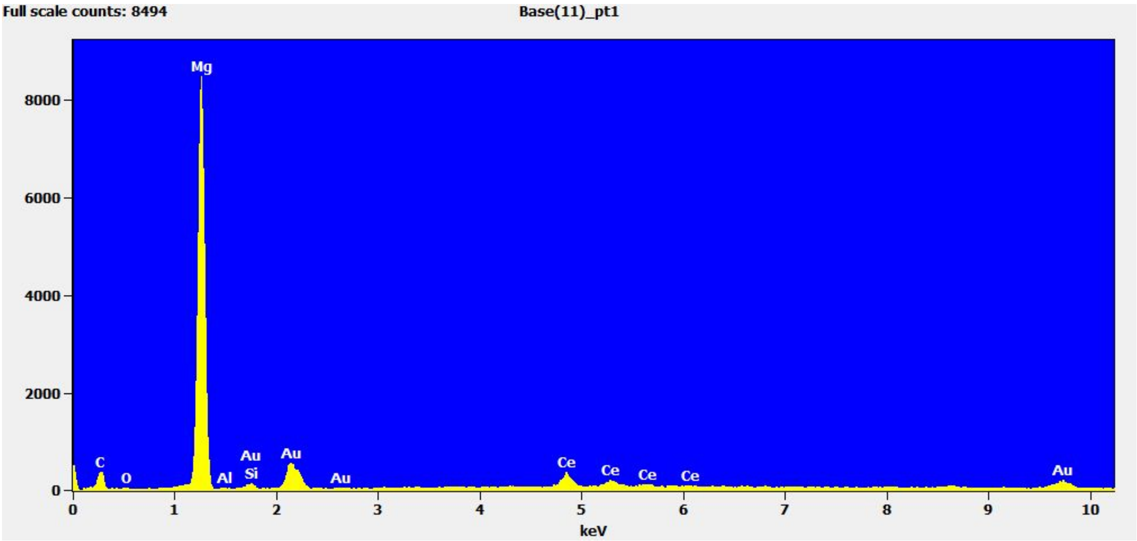

Fig. S1. EDX analysis of the cerium-doped silica mesoporous coating before electrochemical testing.

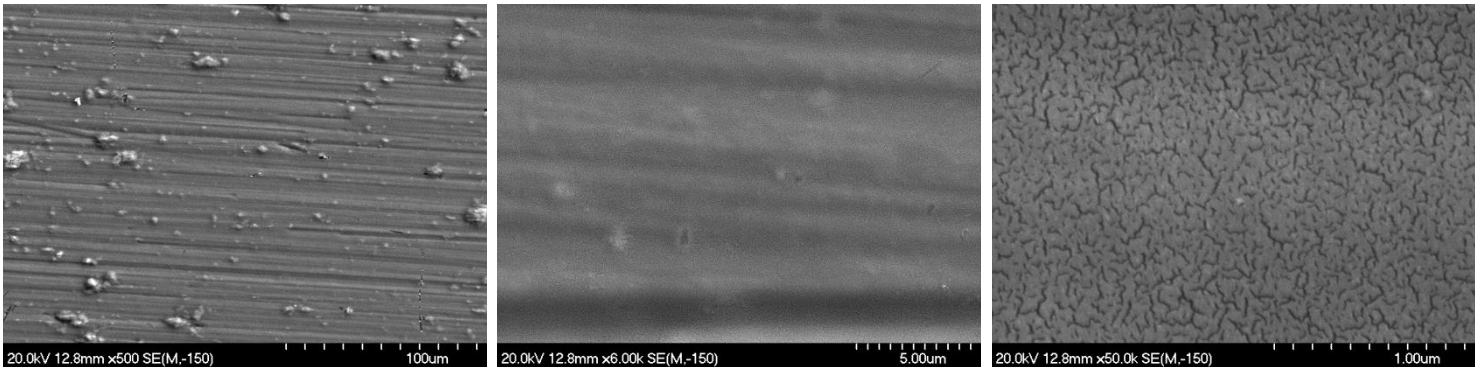

Fig. S2. SEM images of the fresh surface of cerium-doped silica mesoporous coating.

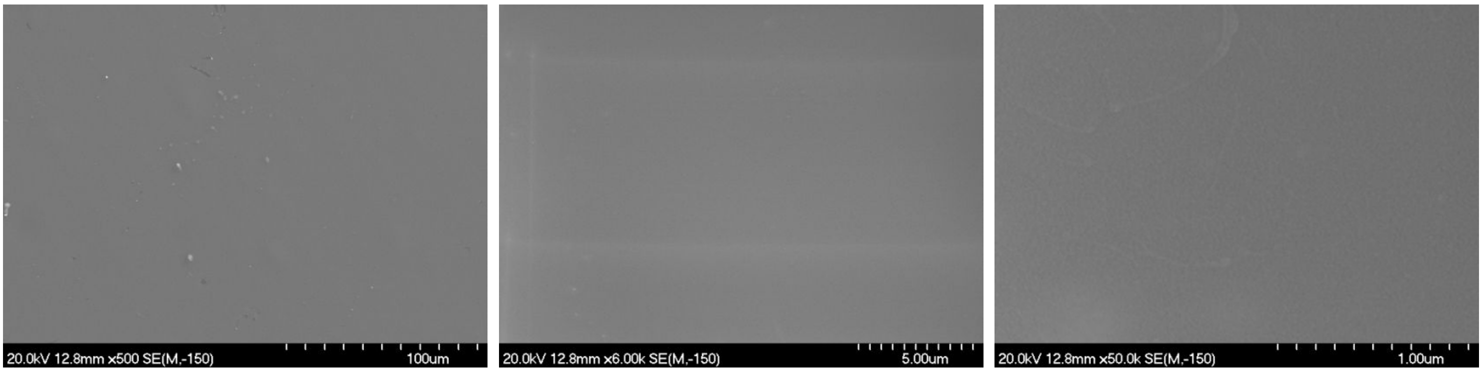

Fig. S3. SEM images of the fresh surface of multilayer coating.

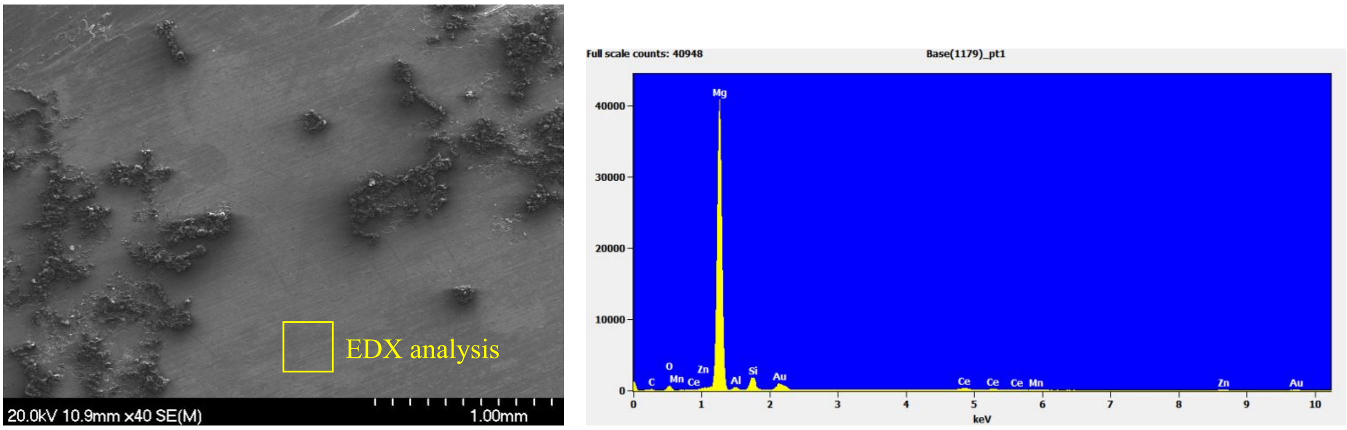

Fig. S4. The SEM image and EDX analysis of the cerium-doped silica mesoporous coating in an area far from corrosion sites (after electrochemical testing).

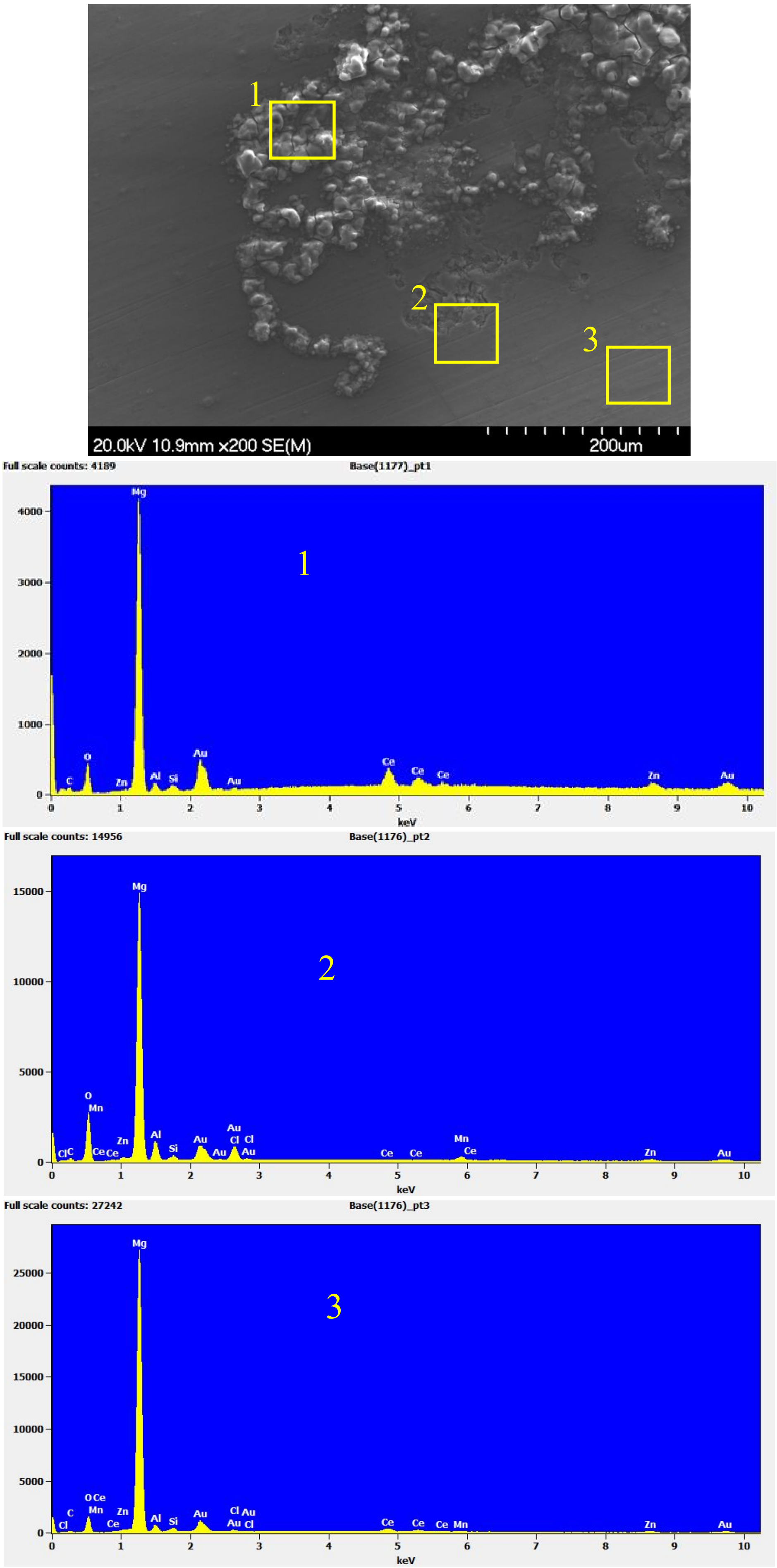

Fig. S5. SEM-EDX analysis of corrosion products on the surface of the cerium-doped silica mesoporous coating (after electrochemical testing).

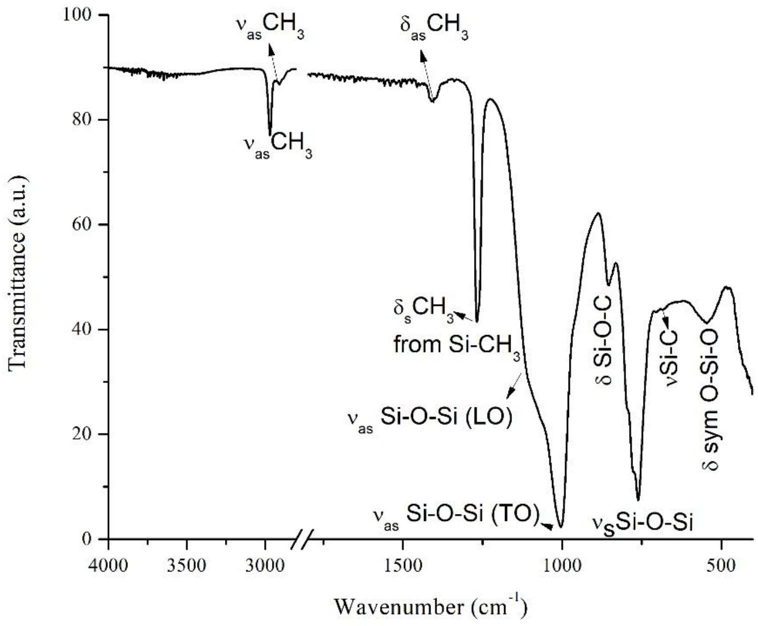

Fig. S6. FTIR analysis of the multilayer coating surface.

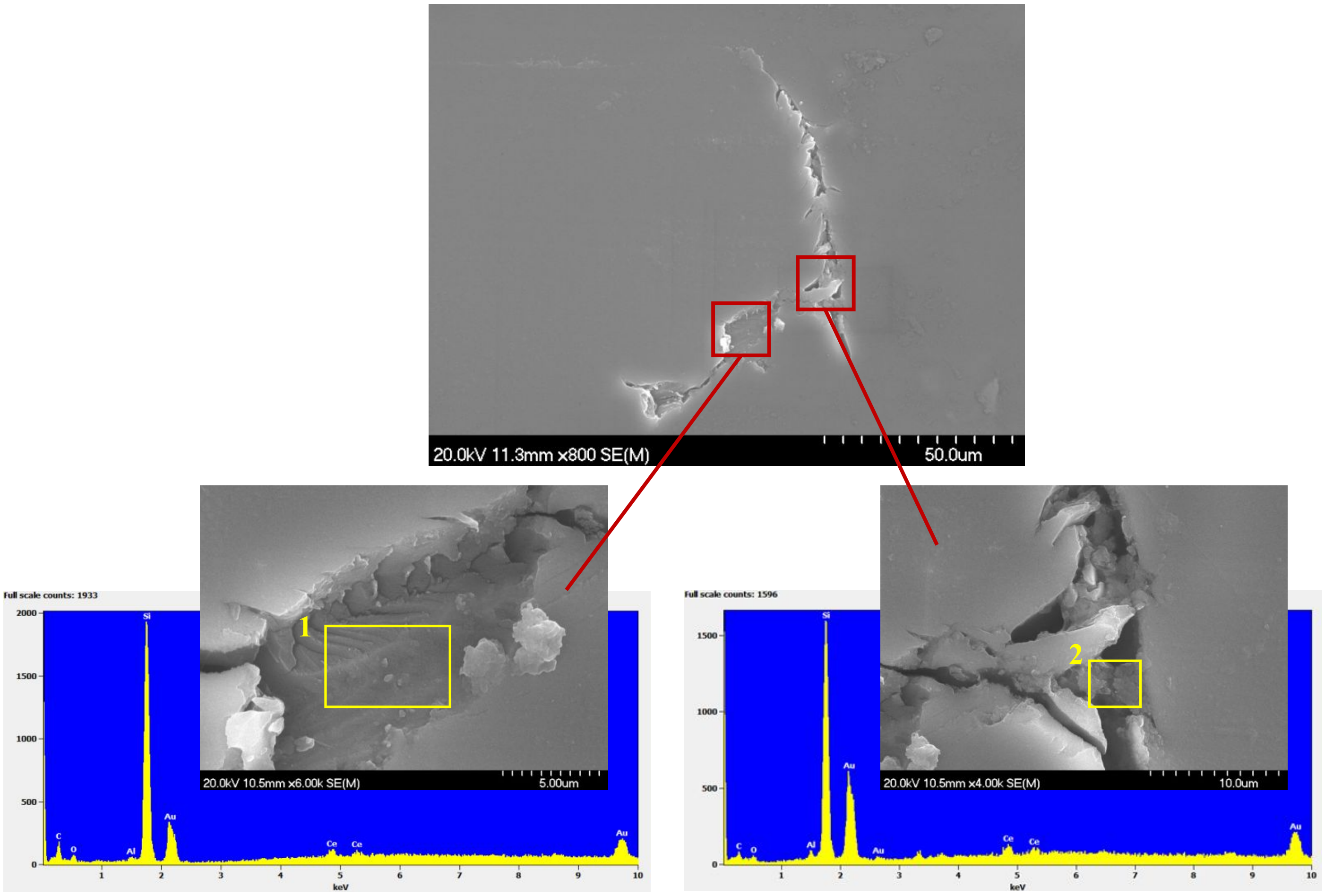

Fig. S7. SEM-EDX analysis of multilayer coating cracks after electrochemical testing.

Table S1. Element concentration from EDX analysis on surface and cross-section of fresh and post-mortem samples.

		Concentration (atom %)								
		C	O	Mg	Si	Cl	Mn	Zn	Ce	Al
Ce layer<br>Fresh – cross section	Ce layer		50.8	46.8	1.0				0.9	0.4
Ce layer<br>Post-mortem - surface	Outside corrosion area	30.5	16.7	47.5	4.8		0.1	0.2	0.2	
	Corrosion products	21.9	53.9	23.8	0.1	0.2	0.1	0.1	0.1	
	Around corrosion products	23.3	47.4	27.7	0.4	0.8	0.2	0.2	0.1	
	Around corrosion products	29.5	30.6	38.4	0.9	0.1	0.1	0.2	0.2	
Multilayer<br>Fresh – cross section	Hybrid layer	21.5	66.5	0,5	11,5					
	Ce layer	10.1	60.9	16.8	11.5				0.4	0.3
	Mg alloy		50.2	48.8						0.9
Multilayer<br>Post-mortem - surface	Crack	84.7	8.2		6.9				0.2	
	Crack	73.8	12.1		13.5				0.6	
